# Comparison Between Effects of Four Crystalloid Solutions on Acid-Base and Electrolyte Abnormalities in Stranded Juvenile Loggerhead Sea Turtles (*Caretta caretta*)

**DOI:** 10.3389/fvets.2022.855744

**Published:** 2022-05-13

**Authors:** Alicia Inurria, Ángelo Santana, Ana B. Casal, Pascual Calabuig, Alejandro Suárez-Pérez, Jorge Orós

**Affiliations:** ^1^Department of Morphology, Veterinary Faculty, University of Las Palmas de Gran Canaria, Las Palmas, Spain; ^2^Department of Mathematics, University of Las Palmas de Gran Canaria, Las Palmas, Spain; ^3^Fuerteventura Sea Turtle Rehabilitation and Conservation Center (Cabildo de Fuerteventura), Centro Veterinario Sur, Pájara, Spain; ^4^Tafira Wildlife Rehabilitation Center (Cabildo de Gran Canaria), Las Palmas, Spain; ^5^La Tahonilla Wildlife Rehabilitation Center (Cabildo de Tenerife), San Cristóbal de La Laguna, Spain

**Keywords:** *Caretta caretta*, fluid therapy, homeostasis, loggerhead, sea turtle

## Abstract

Dehydration, electrolyte abnormalities, and acid-base alterations are common findings in stranded sea turtles. Fluid therapy is essential for reestablishment of homeostasis. The aim of this study was to compare the efficacy and effects on acid-base and electrolyte status of four different crystalloids (0.9% NaCl solution, 0.9% NaCl and lactated Ringer's solutions 1:1 ratio, Plasmalyte, and Jarchow's solution) in 63 stranded juvenile loggerhead turtles (*Caretta caretta*). Crystalloid fluids were administered intracoelomically on the day of admission for a duration of three consecutive days at a rate of 20 mL/kg/day through the inguinal fossa. Blood samples were collected at three timepoints: on admission, 24 h after discontinuing fluid therapy and prior to release. Samples were analyzed using a portable electronic blood analyzer for pH, pCO_2_, pO_2_, bicarbonate, lactate, sodium, potassium, chloride, glucose, and BUN concentration. Thirty-four loggerhead turtles (53.9%) had some type of acid-base alteration at the time of admission. The combination of 0.9% NaCl and lactated Ringer's solutions resulted in the highest percentage of improved/resolved acid-base and electrolyte abnormalities (33.4 % more animals with normal acid-base status compared to the admission time) compared to Jarchow's solution, which was the least effective (15.8% decrease in the number of animals with normal acid-base status compared to the admission time). This study constitutes the second controlled study of fluid therapy in sea turtles, and corroborates the recommendation made previously on the use of 0.9% NaCl + lactated Ringer solution to resolve mild to moderate acid-base alterations in juvenile loggerhead turtles. In addition, convalescent acid-base, electrolyte and plasma biochemical reference intervals are also provided as a standard profile for sea turtle rehabilitation centers.

## Introduction

The loggerhead turtle (*Caretta caretta*) is an endangered species commonly found on the Canary Islands, because this location is in their migration routes. Stranding of loggerhead turtles is common for the Canary Islands (1), and diseases and causes of mortality and/or stranding among loggerhead turtles stranded in their coasts have been described (2–4). Causes of anthropogenic origin are the most frequent (72%), including entanglement in fishing nets and/or plastics (51%), ingestion of hooks and monofilament lines (12%), boat strikes (5%), crude oil (3%), and ingestion of plastics (1%) (4). Dehydration, electrolyte abnormalities, and alterations of acid-base homeostasis are common findings in many of these stranded animals, as evidenced the retrospective study carried out on 66 stranded juvenile loggerhead turtles, in which it was observed that acid-base disorders were present in 86.36% of turtles at the time of admission to our rehabilitation facility (1).

Most studies on metabolic and respiratory alterations in sea turtles have been focused on cold-stunned Kemp's ridley turtles (*Lepidochelys kempii*) (5–7); hyperkalemia, low venous pH, low venous pO_2_, low bicarbonate concentration, and an elevated venous pCO_2_ usually carry a poor prognosis in these turtles (6, 7). In a study on captured loggerhead turtles, pound nets and trawls caused alterations in blood gas, acid-base, and lactate status, though alterations were greater in trawl captured turtles (8). It has also been reported that severe lesions in the salt glands can cause stranding and/or death of loggerhead turtles due to high plasma concentrations of sodium and chloride (9).

Studies on acid-base and electrolyte status of stranded loggerhead turtles are scarce (1, 8, 10, 11). The effects of several crystalloid fluids on the acid-base and plasma biochemical values in stranded juvenile loggerhead turtles in the Canary Islands were reported (12), being the only controlled study published in reptiles. Authors used four different crystalloid fluids: 0.9% NaCl solution, 5% dextrose and 0.9% NaCl solutions 1:1 ratio, 0.9% NaCl and lactated Ringer's solutions 1:1 ratio, and lactated Ringer's solution. The highest percentage of acid-base recovery and electrolyte balance was detected in loggerhead turtles treated with mixed saline-lactated Ringer's solution followed by 0.9% NaCl solution, lactated Ringer's solution, and 5% dextrose and 0.9% NaCl solutions 1:1 ratio (12).

Expanding on our previous work, the aim of this new study was to compare the efficacy and effects on acid-base and electrolyte status of different crystalloid fluids in stranded juvenile loggerhead turtles. The due to their poor performance in improving acid-base and electrolyte abnormalities, the lactated Ringer's solution and dextrose-saline solutions from the previous study were replaced with Plasmalyte (a commercial isotonic crystalloid fluid buffered with gluconate and acetate) (13) and Jarchow's solution, containing 5% dextrose, lactated Ringer's solution and 0.9% NaCl solution in a 1:1:1 ratio (14, 15). Our hypothesis was that Plasmalyte could present a high efficacy, while Jarchow's solution, given its composition, could present similar results to the mixed dextrose-saline solution (12).

## Materials and Methods

### Animals

A total of 63 loggerhead turtles that stranded between April 2017 and September 2021 and were admitted to the Tafira Wildlife Rehabilitation Center or the Fuerteventura Sea Turtle Rehabilitation and Conservation Center were included in this study. No visualization of the gonads was performed. Median (lower quartile, upper quartile; range) straight carapace length (SCL) of the turtles included in this study was 38 (31, 47; 19–56) cm, and weight was 8.8 (6.3, 16.9; 1.1–46.6) kg. Based on SCL, all specimens were identified as juvenile (16). The causes of stranding were entanglement in derelict fishing gear and/or plastics (*n* = 42; 66.6%), ingestion of hooks and monofilament lines (*n* = 6; 9.5%), traumatic injuries caused by boat strikes (*n* = 4; 6.3%), buoyancy disorders (*n* = 2; 3.2%), malnutrition (*n* = 1; 1.6%), and unidentified causes (*n* = 8; 12.7%). Median (lower quartile, upper quartile; range) length of stay in the rehabilitation centers was 81 (53, 137; 18–210) days.

### Fluid Administration

Four groups were established depending on the crystalloid fluid administered. Group 0.9% NaCl solution (*n* = 13); group 0.9% NaCl + lactated Ringer's solutions 1:1 ratio (*n* = 12); group Plasmalyte (Baxter, Deerfield, IL) (*n* = 10); group Jarchow's solution (*n* = 15). An untreated control group (*n* = 13) was also included. Each time a loggerhead turtle arrived at the rehabilitation center, it was assigned to one of the five groups (including the control group) in a cyclic order, regardless of disease severity. Crystalloid fluids were administered intracoelomically on the day of admission for a duration of three consecutive days at a rate of 20 mL/kg/day through the inguinal fossa (17–19). When necessary, other surgical and/or medical procedures were performed. Turtles were transferred to individual outdoor pools with continuous flow of filtered sea water. Data were collected daily, including clinical status, physical activity, food ingestion, cloacal temperature, and weight (19, 20).

Venous blood (0.2 ml) was obtained from the cervical sinus using a nonheparinized syringe. Samples were immediately analyzed using a portable electronic blood analyzer (i-STAT, Heska, Loveland, CO) and CG4+ cartridges (Heska, Loveland, CO) for pH, pCO_2_, pO_2_, bicarbonate, and lactate concentration, and EC8+ cartridges (Heska, Loveland, CO) for concentrations of sodium, potassium, chloride, glucose, and BUN. All turtles were sampled at three timepoints: (a) on admission, prior to any treatment for evaluating the type of acid-base or biochemical disorder, (b) 24 h after the last fluid therapy dose, and (c) prior to release only if they had not received any drug 7 days prior. The same time intervals were used in the control group. Each turtle was released when the veterinary services of each rehabilitation center evaluated it as clinically healthy (1, 12). The reference intervals for convalescent acid-base and electrolyte values (measured at the third timepoint) were used to assess severity of the abnormalities at admission (first timepoint) and after fluid therapy (second timepoint). A turtle was considered to have a normal acid-base status if all pH, pCO_2_ and bicarbonate concentration were within the ranges of the obtained reference intervals.

Because the portable analyzer i-STAT performs the blood tests at 37°C, cloacal temperature of each turtle was recorded with a digital thermometer (Digi-Sense Thermocouple T, Cole-Parmer Instrument Co, Vernon Hills, IL) and several equations more adequate for sea turtles than the algorithms of the i-STAT were used to correct these parameters. The pH, pCO_2_, and pO_2_ were corrected for the cloacal temperature using the following equations (21) (where Δ*T* = 37°C—cloacal temperature):


$$\begin{array}{lll} \text{Corrected pH} & = & \text{pH }\left(\text{at }37^{{}^{\circ}}\text{C}\right)\;+\;0.014\text{ ΔT}\\ \text{Corrected pC}{\text{O}}_{2} & = & \text{pC}{\text{O}}_{2}\;\left(\text{at }37^{{}^{\circ}}\text{C}\right)\;10^{-0.019\text{ ΔT}}\\ \text{Corrected p}{\text{O}}_{2} & = & \text{p}{\text{O}}_{2}\;\left(\text{at }37^{{}^{\circ}}\text{C}\right)\;10^{-0.0058\text{ ΔT}} \end{array}$$

Corrected ${\text{HCO}}_{3}^{-}$ concentrations were calculated using the Henderson-Hasselbalch equation. The CO_2_ solubility coefficient (αCO_2_) and pK were calculated using specific equations for sea turtles (22).

Anion gap (mmol/l) and osmolality (mOsm/kg) were calculated using the following equations (23):


$$\begin{array}{l} \text{Anion gap = }(\text{sodium concentration}\\ \text{ + potassium concentration}\\ \text{ − }(\text{chloride concentration}\\ \text{ + corrected }{\text{HCO}}_{3}^{-}\;\text{concentration}).\\ \text{Osmolality = 2}(\text{sodium concentration}\\ \text{ + potassium concentration})\\ \text{ + }(\text{glucose concentration/18})\\ \text{ + }(\text{BUN}\;\text{concentration/2}.\text{8}). \end{array}$$

### Statistical Analysis

The statistical analysis was performed using the R statistical environment version 4.1.2 (R Development Core Team, Vienna, Austria). Variables with normal distribution were described as mean and standard deviation. Not normally distributed variables were described as median and quartiles. For every treatment (group), differences between variable values through the study (i.e., at admission, after fluid therapy, and prior to release) were assessed by using repeated measures ANOVA for normally distributed and repeated measures Friedman test form not normally distributed variables. Those cases in which there were significant differences between measures, *post-hoc* tests were carried out using pairwise *t*-tests for normal variables, and pairwise Wilcoxon tests for not normally distributed variables. In both cases, *P*-values were adjusted with Bonferroni method. For comparing baseline characteristics of the loggerhead turtles in the different treatment groups upon admission, and also the effects of the different treatments after fluid therapy, analysis of variance was used for normally distributed variables, and Kruskal-Wallis test for not normally distributed variables. When significant differences were observed, *post-hoc* comparisons were made using Tukey tests for normally distributed variables, and Conover tests for not normally distributed variables. Values of *P* < 0.05 were considered statistically significant.

Reference intervals for convalescent loggerhead turtles were obtained from the pre-release values of convalescent turtles. Each variable was assessed using histograms, boxplots and normal probability plots. Normality was tested by using the Shapiro-Wilk test, and Box Cox transformations were performed on variables that were not normally distributed. For variables that after transformation did not conform to a Gaussian distribution, non-parametric method was used for reference interval estimation. In addition to visual examination of boxplots and histograms, outliers were identified using the Horn outlier detection algorithm (24) and were manually removed from reference interval estimation. The 95% reference intervals were estimated using the robust method following AVSCP reference intervals guidelines (2.5–97.5 percentiles) (25). Associated 90% confidence intervals (CIs) for the limits of each interval were computed by bootstrap methods following same guidelines. All computations were made by using the R package Reference Intervals.

## Results

Thirty-four turtles (53.9%) had some type of mild to moderate acid-base alteration at the time of admission. Of these, thirteen (20.6%) had metabolic and respiratory acidosis, and seven turtles (11.1%) had respiratory acidosis or metabolic acidosis. Of those with metabolic and respiratory acidosis, four turtles had high anion gap values, and the other nine had normal anion gap values with hyperchloremic acidosis. There were no statistically significant differences between treatment groups at baseline.

Fourteen turtles died or were euthanized during their stay in the rehabilitation center after receiving fluid therapy, so that pre-release values could not be obtained; therefore, the reference intervals were calculated based on 49 released turtles. Neither euthanasia nor death during stay in the rehabilitation facilities were overrepresented in any group. Nine additional turtles that had died or had been euthanized between admission and complete fluid therapy, and therefore the biochemical blood values could not be obtained, were excluded from the study.

**Figure 1:**
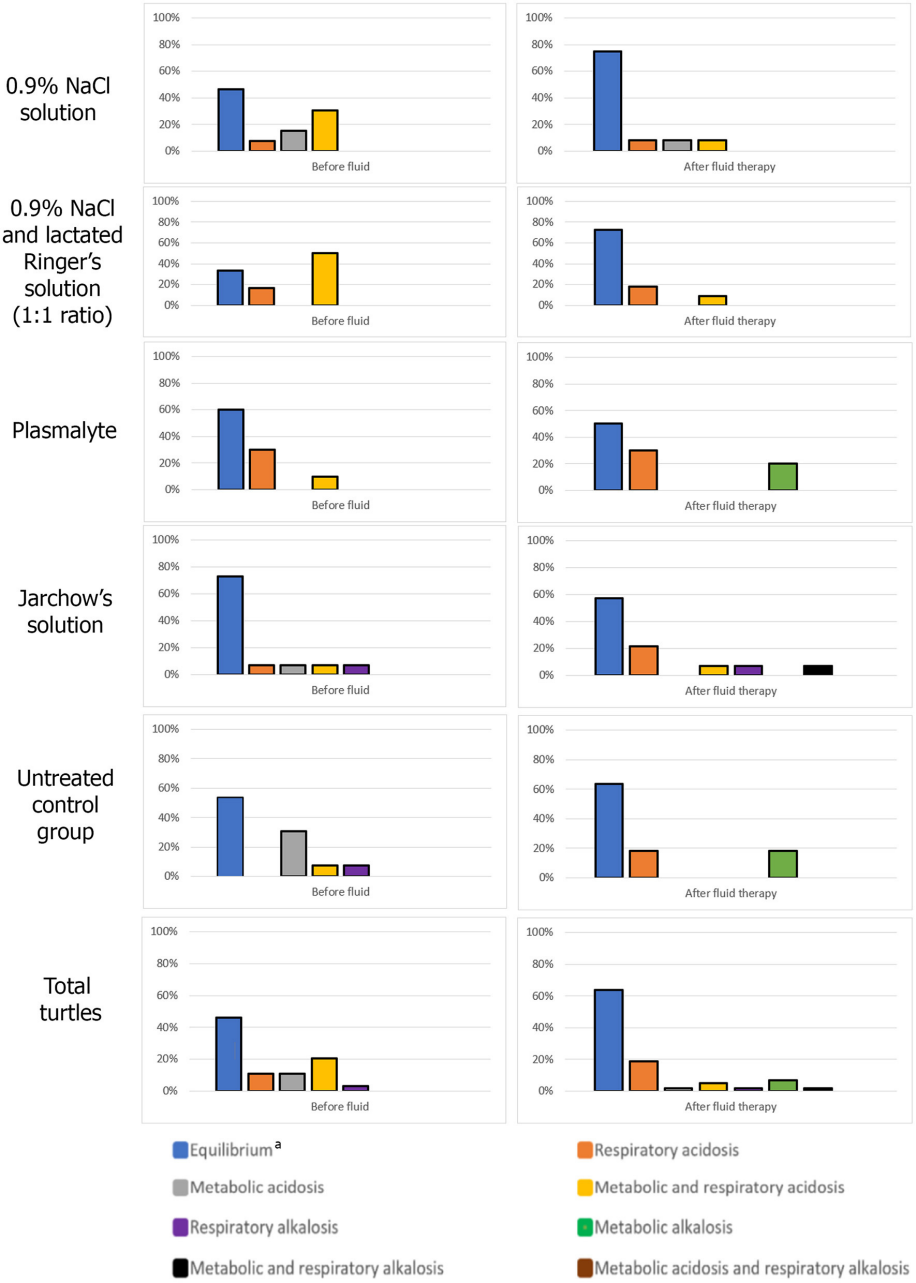
Acid-base status upon admission and after fluid therapy for each loggerhead turtle group. ^a^All pH, pCO_2_ and bicarbonate concentration within the ranges of the obtained reference intervals.

The group treated with 0.9% NaCl + lactated Ringer's solution showed the highest percentage of resolution of acid-base abnormalities (33.4% compared to admission) followed by the group receiving 0.9% NaCl (28.8% compared to admission). In comparison in the control group, only 9.8% of the turtles showed resolution of the acid-base abnormalities (Figure 1). When Plasmalyte and Jarchow's solutions were used, a 10% and 15.8% decrease in the number of animals with normal acid-base status compared to the admission time were observed, respectively. It was observed an increase in the number of turtles with metabolic alkalosis in the Plasmalyte group.

Values of pH, blood gas and plasma biochemical parameters at the three timepoints (on admission, 24 h after fluid therapy, and prior to release) for treated groups and control group are shown in Tables 1, 2, respectively.

**Table 1:** Median (and quartiles), and mean and standard deviation of pH, blood gas and plasma biochemical parameters during times of sampling of the four treatment groups.

	**0.9% NaCl**			**0.9% NaCl** **+** **lactated Ringer solutions (1:1 ratio)**			**Plasmalyte**			**Jarchow's solution**		
	**Admission**	**After fluid therapy**	**Prior to release**	**Admission**	**After fluid therapy**	**Prior to release**	**Admission**	**After fluid therapy**	**Prior to release**	**Admission**	**After fluid therapy**	**Prior to release**
**Not normally distributed**												
**pH**	7.57 (7.49, 7.61)	7.62 (7.55, 7.64)	7.6 (7.55, 7.62)	**7.48^a^** **(7.45, 7.57)**	**7.57^b^** **(7.54, 7.61)**	**7.66^c^** **(7.61, 7.68)**	7.51 (7.5, 7.54)	7.62 (7.59, 7.72)	7.56 (7.55, 7.6)	7.61 (7.5, 7.65)	7.65 (7.61, 7.68)	7.59 (7.56, 7.61)
**pO**_**2**_ (mmHg)	68.63 (63.89, 76.52)	74.6 (67.98, 81.67)	72.58 (61.99, 77.99)	83.3 (74.16, 145.23)	89.7 9 (72.5, 119.44)	72 (68.32, 77.17)	73.26 (59.55, 84.53)	67.19 (60.77, 88.87)	75.93 (61.7, 84.56)	83.46 (72.3, 124.93)	80 (72.87, 109.9)	75.46 (67.6, 96.98)
**Lactate** (mmol/L)	4.79 (1.87, 8.51)	0.85 (0.37, 1.11)	0.75 (0.52, 1.16)	3.42 (2.29, 5.51)	1.11 (0.61, 1.76)	0.98 (0.4, 1.65)	**4.4^a^** **(2.54, 6.96)**	0.71 (0.48, 1.74)	**0.9^c^** **(0.7, 1.98)**	2.72 (1.88, 4.17)	1.35 (1, 1.9)	**1.06^c^** **(0.45, 1.4)**
**Sodium** (mmol/L)	150 (148, 154)	149 (146.5, 151)	151.5 (149.25, 153)	**149^a^** **(147, 151.25)**	**146^b^** **(145, 147.5)**	151 (147.5, 154)	150.5 (146.25, 152)	149 (147, 150.75)	153.5 (150, 154)	150 (148, 152)	**149^b^** **(146.25, 151)**	**152.5^c^** **(151, 156)**
**Potassium** (mmol/L)	**3.5^a^** **(2.7, 3.8)**	**3.1^b^** **(2.55, 3.1)**	3.3 (3.2, 3.63)	3.5 (2.95, 3.87)	**3.4^b^** ** (2.9, 3.8)**	**2.95^c^** **(2.73, 3.15)**	**3.85^a^** **(3.3, 4.07)**	2.95 (2.4, 3.15)	**3.25^c^** **(2.82, 3.6)**	3.2 (2.65, 3.6)	2.7 (2.5, 3.2)	2.8 (2.38, 3.6)
**Anion Gap** (mmol/L)	**7.33^a^** **(5.39, 9.78)**	4.38 (1.2, 7.78)	**3.55^c^** ** (0.91, 4.68)**	**5.45^a^** **(3.31, 11.5)**	0.85 (-0.12, 2.58)	3.18 (0.89, 6.08)	3.89 (-0.9, 5.98)	−0.39 (-3.3, 6.92)	**7.55^c^** **(5.99, 7.71)**	5.69 (3.25, 6.64)	1.82 (0.32, 7.89)	6.2 (3.99, 9.06)
**Glucose** (mg/dL)	102 (87, 115)	100 (90, 129)	112.5 (107.25, 120)	110.5 (83.75, 137.5)	86 (77, 133)	107.5 (92.5, 114.5)	94 (67.25, 105.5)	113 (87.75, 124.5)	98 (85.75, 105)	135 (100.5, 157)	124 (104, 176)	119.5 (113.5, 122.5)
**BUN** **(mg/dL)^d^**	55 (34, 90)	**58^b^** ** (48.5, 71)**	**140^c^** ** (140, 140)**	**50.5^a^** ** (39.5, 60.25)**	**68^b^** ** (55, 72)**	**140^c^** ** (139, 140)**	**71.5^a^** ** (57.25, 105)**	**79.5^b^** ** (66.25, 98.25)**	**138^c^** ** (133, 140)**	**63^a^** ** (48.5, 86)**	**71^b^** ** (50, 112)**	**140^c^** ** (111.75, 140)**
**Normally distributed**												
**pCO**_**2**_ (mmHg)	27.96 ± 5.44	29.23 ± 4.61	29.96 ± 4.65	28.51 ± 5.57	30.91 ± 7.39	28.45 ± 4.85	35.18 ± 8.18	34.18 ± 8.67	29.83 ± 3.64	28.42 ± 6.08	30.62 ± 9.7	30.71 ± 4.78
**HCO**_**3**_ (mmol/L)	**34.88** **±11.79^a^**	38.24 ± 6.2	**38.1** **±3.01^c^**	**31.84** **±8.91^a^**	39.11 ± 12.15	**41.3** **±5.55^c^**	**40.06** **±10.99^a^**	**50.89** **±9.5^b^**	37.49 ± 5.57	**37.66** **±7.26^a^**	**44.25** **±8.4^b^**	40.01 ± 4.78
**Chloride** (mmol/L)	**113.92** **±5.63^a^**	**107.73** **±6.89^b^**	114.1 ± 3.98	**113.42** **±8.12^a^**	107.45 ± 7.57	109.6 ± 6.48	**107.8** **±3.94^a^**	**99.7** **±4.67^b^**	111.17 ± 6.74	**110.6** **±5.63^a^**	**104.08** **±6.44^b^**	109.67 ± 6.33
**Osmolality** (mOsmol/kg)^d^	338.09 ± 20.54	**333.04** **±11.64^b^**	**365.58** **±7.6^c^**	328.05 ± 8.71	**324.61** **±14.18^b^**	**360.65** **±9.87^c^**	336.54 ± 26.77	**339.48** **±14.97^b^**	**364.58** **±7.85^c^**	338.3 ± 14	**337.62** **±19.29^b^**	**361.55** **±13^c^**

a *Statistically significant differences between admission and after fluid therapy values within each group*;

b *Statistically significant differences between after fluid therapy values and prior to release values within each group*;

c *Statistically significant differences between admission and prior to release values within each group*;

d *Osmolality and BUN values obtained prior to release were underestimated because BUN values exceeded in many turtles the analytical range of the analyzer (140 mg/dL). Bold values indicate with statistically significant differences*.

**Table 2:** Median (and quartiles), and mean and standard deviation of pH, blood gas and plasma biochemical parameters during times of sampling of untreated turtles (control group).

	**Admission**	**Control day**	**Prior to release**
**Not normally distributed**			
**pH**	7.65 (7.51, 7.7)	7.68 (7.65, 7.71)	7.69 (7.58, 7.73)
**pO**_**2**_ (mmHg)	109.09 (92.19, 126.31)	90.12 (74.14, 135.2)	82.16 (75.81, 104.18)
**Lactate** (mmol/L)	**4.36**^**a**^ (2.8, 7.01)	**1.93**^**b**^ (0.84, 2.17)	**1.09**^**c**^ (0.47, 1.42)
**Sodium** (mmol/L)	149 (147, 150)	150 (147.5, 152)	151 (149.25, 156)
**Potassium** (mmol/L)	**3.7**^**a**^ (3.4, 3.7)	**3.2**^**b**^ (2.8, 3.3)	2.75 (2.52, 3.18)
**Anion Gap** (mmol/L)	**7.33**^**a**^ (6, 10.37)	−0.59 (-2.53, 1.11)	5.13 (4.56, 5.66)
**Glucose** (mg/dL)	99 (82, 110)	98 (90, 117)	107 (99, 123.25)
**BUN** (mg/dL)^d^	**59**^**a**^ (47, 93)	**108**^**b**^ (71.5, 120.5)	**140**^**c**^ (140, 140)
**Normally distributed**		
**pCO**_**2**_ (mmHg)	**23.79** **±5.86**^**a**^	29.37 ± 4.65	26.20 ± 4.46
**HCO**_**3**_ (mmol/L)	**32.57** **±8.13**^**a**^	**47.34** **±8.83**^**b**^	**40.29** **±5.72**^**c**^
**Chloride** (mmol/L)	**113.31** **±5.23**^**a**^	**104.82** **±6.87**^**b**^	110.2 ± 6.97
**Osmolality** (mOsmol/kg)^d^	**336.79** **±17.79**^**a**^	**345.19** **±17.3**^**b**^	**365.09** **±12.07**^**c**^

*^a^Statistically significant differences between admission and control day values; ^b^Statistically significant differences between control day values and prior to release values; ^c^Statistically significant differences between admission and prior to release values. ^d^Osmolality and BUN values obtained prior to release were underestimated because BUN values exceeded in many turtles the analytical range of the analyzer (140 mg/dL). Bold values indicate statistically significant differences*.

There were several statistically significant differences between admission values and those values obtained after therapy and just prior to release. Initial pH values were significantly lower (*P* = 0.0017) than post-therapy values in the group treated with 0.9% NaCl + lactated Ringer's solution. Bicarbonate concentrations were significantly higher after fluid therapy compared to the admission concentrations in all treated groups (*P* < 0.0247), and were significantly higher prior to release compared to the admission concentrations in groups 0.9% NaCl solution (*P* = 0.0247) and 0.9% NaCl + lactated Ringer's solutions (*P* = 0.0082). Post-therapy lactate concentrations were lower than those obtained on admission, but only statistically significant (*P* = 0.011) in the group treated with Plasmalyte. Post-therapy sodium concentrations were lower than initial concentrations in all treated groups, but these differences were statistically significant (*P* = 0.02) only in the group treated with 0.9% NaCl + lactated Ringer's solution. Post-therapy chloride concentrations were significantly lower than those obtained on admission in all groups (*P* < 0.025). Potassium concentrations were lower after fluid therapy compared to the admission concentrations in all treated groups, but only statistically significant in the groups treated with 0.9% NaCl (*P* = 0.0498) and Plasmalyte (*P* = 0.03). Although differences in glucose concentrations after treatment compared to initial concentrations were not significant in any group, post-therapy hyperglycemia was observed in 69.2% of the turtles treated with Jarchow's solution.

Reference intervals and 90% confidence intervals for portable blood gas analyzer (i-STAT) parameters in convalescent loggerhead sea turtles are presented in Table 3.

**Table 3:** Reference intervals and 90% confidence intervals for portable blood gas analyzer (i-STAT) parameters in convalescent loggerhead sea turtles.

	**Median (range)**	**Lower limit (90%CI)**	**Upper limit (90% CI)**
**pH**	7.60 (7.51–7.77)	7.47 (7.49–7.44)	7.76 (7.80–7.72)
**pCO**_**2**_ (mmHg)	29.38 (17.29–42.52)	21.68 (19.84–23.38)	37.03 (35.72–38.49)
**pO**_**2**_ (mmHg)	75.91 (50.93–198.07)	51.72 (55.00–48.43)	129.83 (153.61–113.26)
**HCO**_**3**_ (mmol/L)	39.52 (29.99–51.64)	29.32 (27.20–31.17)	49.47 (47.32–51.61)
**Lactate** (mmol/L)	0.89 (0.30–6.72)	0.23 (0.28–0.18)	7.34 (13.13–4.53)
**Sodium** (mmol/L)	151.50 (143.00–160.00)	144.29 (142.67–145.67)	159.68 (158.21–161.34)
**Potassium** (mmol/L	3.00 (2.10–4.10)	1.99 (1.80–2.18)	4.07 (3.89–4.27)
**Chloride** (mmol/L)	110.50 (98.00–122.00)	98.35 (95.91–100.58)	123.39 (121.02–126.12)
**Anion Gap** (mmol/L)	4.70 (−8.24 to 14.07)	−1.37 (−2.85 to −0.03)	11.42 (10.19–12.79)
**Glucose** (mg/dL)	110.00 (70.00–152.00)	77.33 (69.23–85.30)	143.84 (137.17–150.10)
**Osmolality** (mOsmol/kg)	364.58 (333.91–381.27)	341.5-378.22	345.62-383.7

## Discussion

Over half of stranded loggerhead turtles (53.9%) exhibited some type of mild to moderate acid-base alteration at the time of admission, which is consistent with that reported in previous studies on juvenile loggerhead turtles stranded in the Canary Islands (1, 12). Other studies, particularly on cold-stunned turtles showed more extreme acid-base derangements (6, 11). Metabolic acidosis is very common in stranded sea turtles, being associated with an increased production of acid or loss of base (1); potential causes of acid gain include entanglement in fishing nets, involuntary submergence, shock, and renal disorders (1). Among the causes of loss of base are digestive disorders (e.g., diarrhea), and renal disorders (26). Respiratory acidosis occurs due to CO_2_ retention, as seen with hypoventilation, upper or lower airway obstruction (such as bronchopneumonia) or hypoxia (27).

The choice of the appropriate fluid to administer to the turtle should be made according to the condition of each individual (18), but these analytical determinations are not always possible, especially in rehabilitation centers with limited material and human resources. Crystalloid fluids containing gluconate, acetate and lactate contribute to increasing alkalinity thus counteracting acidosis (28). In the present study, the crystalloid solutions with the worst results in the 2015 study (lactated Ringer's solution and dextrose-saline solutions) were replaced by two different solutions: Plasmalyte and Jarchow's solution. The solutions with the best results in the 2015 study (mixed saline-lactated Ringer's solution, and saline solution) were maintained in order to corroborate the results of the previous study, and also comply with the guidelines of the Ethical Committee for Animal Experimentation. Because the study involved clinical actions on animals with special protection, it was necessary to guarantee, in addition to investigating possible treatments, the highest possible survival rate. As in the previous study (12), the greatest increase in the number of animals with normal acid-base status compared to the admission time after 3 days of therapy was observed when the mixed saline-lactated Ringer's solution was used. Although several authors suggested that lactated crystalloids may exacerbate hyperlactatemia in reptiles (29, 30), concerns that its use may be harmful to reptiles have not been validated (13). Lactate is rapidly converted to bicarbonate in the liver, and its use is recommended unless there is concern for hepatic dysfunction (31).

Treatment with Jarchow's solution presented the worst results restoring the acid-base balance, as was observed in the previous study (12) when a similar solution containing 1:1 5% dextrose + 0.9% NaCl was administered. Administering this dextrose solution to a hypovolemic patient would not only fail to expand the intravascular space and restore acid-base balance, but would also lead to extravasation, edema, altered oxygen transportation to the tissues and therefore potentially exacerbate acid-base abnormalities. Furthermore, similarly to the study in 2015 (12), the highest post-therapy glucose levels were observed when Jarchow's solution was administered, due to its dextrose content. Recently, administration of Jarchow's solution for rehydration of experimentally dehydrated bearded dragons (*Pogona vitticeps*) resulted in severe hyperglycemia and significant reductions in plasma osmolarity and sodium and phosphorus concentrations (32).

Results after Plasmalyte treatment were unexpected. Plasmalyte is commonly used in reptile medicine (32), especially as its buffers (gluconate and acetate) are not liver dependent due to their pharmacokinetics (30). Gluconate produces bicarbonate in peripheral tissues and muscles, but excessive administration of this compound may lead to metabolic alkalosis and hypokalemia (30). When alkalosis occurs, there is a gastrointestinal and renal compensatory hydrogen loss, which also produces electrolyte loss that may lead to hypokalemia (33). Considering that most of the turtles in this study had mild to moderate acid-base abnormalities, Plasmalyte might have overcorrected those. Therefore, Plasmalyte might be only appropriate to correct severe acid-base abnormalities and it should be reserved for those cases.

The present study had several limitations when evaluating clinical fluid therapy for loggerhead turtles. Although its use is widespread in rehabilitation centers around the world, the portable electronic blood analyzer i-STAT is not validated for use in reptiles. In addition, hepatic, pancreatic, and complete renal panels were not performed in this study. It would have been useful to know how possible disorders of these organs could influence the response to fluid therapy. And finally, there were several additional factors (cause of stranding and associated diseases, medical treatments received, and length of stay in the rehabilitation facilities) that could have influenced the results of our study.

In conclusion, up to 53.9% of the stranded loggerhead turtles of this study presented a mild to moderate acid-base alteration. The highest percentage of acid-base and electrolyte balance after 3 days of therapy was observed when the mixed saline-lactated Ringer's solution was used, which corroborates a previous study in which other solutions were administered (12). Furthermore, establishment of convalescent acid-base, electrolyte and plasma biochemical reference intervals could be useful for rehabilitation centers caring for stranded loggerhead turtles.

## Data Availability Statement

The original contributions presented in the study are included in the article/supplementary material, further inquiries can be directed to the corresponding author/s.

## Ethics Statement

The animal study was reviewed and approved by Ethical Committee for Animal Experimentation (CEEA-ULPGC).

## Author Contributions

AI and JO: conceptualization, methodology, investigation, data curation, and write-up (original draft). ÁS: statistical analysis. AC, PC, and AS-P: methodology and data curation. All authors contributed to the article and approved the submitted version.

## Funding

This research received external funding from the national project CGL2015-69084-R (Ministry of Economic Affairs and Digital Transformation MINECO, Spain Government/European Regional Development Fund FEDER, European Commission).

## Conflict of Interest

The authors declare that the research was conducted in the absence of any commercial or financial relationships that could be construed as a potential conflict of interest.

## Publisher's Note

All claims expressed in this article are solely those of the authors and do not necessarily represent those of their affiliated organizations, or those of the publisher, the editors and the reviewers. Any product that may be evaluated in this article, or claim that may be made by its manufacturer, is not guaranteed or endorsed by the publisher.

## References

[1] Camacho M Quintana MP Luzardo OP Estévez MD Calabuig P Orós J. Metabolic and respiratory status of stranded juvenile loggerhead sea turtles (*Caretta caretta*): 66 cases (2008–2009). J Am Vet Med Assoc. (2013) 242:396–401. 10.2460/javma.242.3.396 23327184

[2] Orós J Calabuig P Monagas P Casal AB Déniz S. Digestive pathology of sea turtles stranded in the Canary Islands between 1993 and 2001. Vet Rec. (2004) 155:169–74. 10.1136/vr.155.6.169 15357377

[3] Orós J Torrent A Calabuig P Déniz S. Diseases and causes of mortality among sea turtles stranded in the Canary Islands, Spain (1998-2001). Dis Aquat Org. (2005) 63:13–24. 10.3354/dao063013 15759796

[4] Orós J Montesdeoca N Camacho M Arencibia A Calabuig P. Causes of stranding and mortality, and final disposition of loggerhead sea turtles (*Caretta caretta*) admitted to a wildlife rehabilitation center in Gran Canaria Island, Spain (1998–2014): a long-term retrospective study. PLoS One. (2016) 11:e0149398. 10.1371/journal.pone.0149398 26901623 PMC4763070

[5] Innis CJ Tlusty M Merigo C Weber ES. Metabolic and respiratory status of cold stunned Kemp's ridley sea turtles (*Lepidochelys kempii*). J Comp Physiol B. (2007) 177:623–30. 10.1007/s00360-007-0160-9 17431640

[6] Keller KA Innis CJ Tlusty MF Kennedy AE Bean SB Cavin JM. Metabolic and respiratory derangements associated with death in cold-stunned Kemp's ridley turtles (*Lepidochelys kempii*): 32 cases (2005–2009). J Am Vet Med Assoc. (2012) 240:317–23. 10.2460/javma.240.3.317 22256849

[7] Stacy NI Innis CJ Hernandez JA. Development and evaluation of three mortality prediction indices for cold-stunned Kemp's ridley sea turtles (*Lepidochelys kempii*). Conserv Physiol. (2013) 1:cot003. 10.1093/conphys/cot003 27293587 PMC4732445

[8] Harms CA Mallo KM Ross PM Segars A. Venous blood gases and lactates of wild loggerhead sea turtles (*Caretta caretta*) following two capture techniques. J Wildl Dis. (2003) 39:366–74. 10.7589/0090-3558-39.2.366 12918445

[9] Orós J Camacho M Calabuig P Arencibia A. Salt gland adenitis as only cause of stranding of loggerhead sea turtles *Caretta caretta*. Dis Aquat Org. (2011) 95:163–6. 10.3354/dao02351 21848124

[10] Chittick EJ Stamper MA Beasley JF Lewbart GA Horne WA. Medetomidine, ketamine, and sevoflurane for anesthesia of injured loggerhead sea turtles: 13 cases (1996–2000). J Am Vet Med Assoc. (2002) 221:1019–25. 10.2460/javma.2002.221.1019 12369681

[11] Innis CJ McGowan JP Burgess EA. Cold-stunned loggerhead sea turtles (*Caretta caretta*): initial vs. convalescent physiologic status and physiologic findings associated with death. J Herpetol Med Surg. (2019) 29:105–12. 10.5818/19-06-204.1

[12] Camacho M Quintana MP Calabuig P Luzardo OP Boada LD Zumbado M. Acid-base and plasma biochemical changes using crystalloid fluids in stranded juvenile loggerhead sea turtles (*Caretta caretta*). PLoS ONE. (2015) 10:e0132217. 10.1371/journal.pone.0132217 26167930 PMC4500549

[13] Petritz OA Son TT. Emergency and critical care. In: Divers SJ, Stahl SJ, editors. Mader's Reptile and Amphibian Medicine and Surgery 3rd edition. St. Louis, MO: Elsevier (2019). p. 967–76. 10.1016/B978-0-323-48253-0.00087-8

[14] Mader DR Rudloff E. Emergency and critical care. In: Mader DR, editor. Reptile Medicine and Surgery 2nd edition. St. Louis, MO: Saunders Elsevier (2006). p. 533–48. 10.1016/B0-72-169327-X/50035-3

[15] Wellman ML DiBartola SP Kohn CW. Applied physiology of body fluids in dogs and cats. In: DiBartola S, editor. Fluid, Electrolyte, and Acid-Base Disorders in Small Animal Practice 3rd edition. St. Louis, MO: Saunders Elsevier (2006). p. 3–26. 10.1016/B0-72-163949-6/50004-7

[16] Casale P Mazaris AD Freggi D. Estimation of age and maturity of loggerhead sea turtles *Caretta caretta* in the Mediterranean using length-frequency data. Endanger Species Res. (2011) 13:123–9. 10.3354/esr00319

[17] Norton TM Walsh MT. Sea turtle rehabilitation. In: Miller RE, Fowler ME, editors. Fowler's Zoo and Wild Animal Medicine. Current Therapy Vol. 7. St. Louis, MO: Elsevier Saunders (2012). p. 239–46. 10.1016/B978-1-4377-1986-4.00031-7

[18] Innis CJ Harms CA Manire CA. Therapeutics. In: Manire CA, Norton TM, Stacy BA, Innis CJ, Harms CA, editors. Sea Turtle Health & Rehabilitation. Plantation, FL: J. Ross Publishing (2017). p. 497–526.

[19] Innis CJ. Medical management and rehabilitation of sea turtles. In: Divers SJ, Stahl SJ, editors. Mader's Reptile and Amphibian Medicine and Surgery 3^rd^ edition. St. Louis, MO: Elsevier (2019). p. 1382–8. 10.1016/B978-0-323-48253-0.00176-8

[20] Norton TM. Chelonian emergence and critical care. Semin Avian Exot Pet. (2005) 14:106–30. 10.1053/j.saep.2005.04.005

[21] Ashwood ER Kost G Kenny M. Temperature correction of blood-gas and pH measurements. Clin Chem. (1983) 29:1877–85. 10.1093/clinchem/29.11.1877 6354511

[22] Stabenau EK Heming TA. Determination of the constants of the Henderson-Hasselbalch equation, (CO[[sb]]2[[/s]] and pKa, in sea turtle plasma. J Exp Biol. (1993) 180:311–4. 10.1242/jeb.180.1.311

[23] Michell AR Bywater RJ Clarke KW Hall LW Waterman AE. Clinical and laboratory assessment of deficits and disturbances. In: Michell AR, Bywater RJ, Clarke KW, Hall LW, Waterman AE, editors. Veterinary Fluid Therapy. Oxford: Blackwell Scientific Publications (1989). p. 55–103.

[24] Horn PS Feng L Li Y Pesce AJ. Effect of outliers and nonhealthy individuals on reference interval estimation. Clin Chem. (2001) 47:2137–45. 10.1093/clinchem/47.12.2137 11719478

[25] Friedrichs KR Harr KE Freeman KP Szladovits B Walton RM Barnhart KF. reference interval guidelines: determination of de novo reference intervals in veterinary species and other related topics. Vet Clin Pathol. (2012) 41:441–53. 10.1111/vcp.12006 23240820

[26] Espino-López L Torío-Álvarez R Goicoa-Valdevira A. Valoración clínica y laboratorial de los desequilibrios hidroelectrolíticos y ácido-básicos. In: Rejas-López J, Fidalgo-Álvarez LE, Goicoa-Valdevira A, González-Montaña JR, editors. Aplicaciones de Fluidos en Veterinaria. Valencia: Consulta de Difusión Veterinaria SL (2001). p. 35–52.

[27] Tuñón-González MJ. Bases fisiológicas de la fluidoterapia. In: Rejas-López J, Fidalgo-Álvarez LE, Goicoa-Valdevira A, González-Montaña JR, editors. Aplicaciones de Fluidos en Veterinaria. Valencia: Consulta de Difusión Veterinaria SL (2001). p. 7–32.

[28] DiBartola SP Bateman S. Introduction to fluid therapy. In: DiBartola S, editor. Fluid, Electrolyte, and Acid-Base Disorders in Small Animal Practice 3rd edition. St. Louis, MO: Saunders Elsevier (2006). p. 325–43. 10.1016/B0-72-163949-6/50017-5

[29] Wilkinson R. Therapeutics. In: McArthur S, Wilkinson R, Meyer J, editors. Medicine and Surgery of Tortoises and Turtles. Oxford: Blackwell Publishing Ltd. (2004). p. 481–3.

[30] Mitchell MA. Therapeutics. In: Mader DR, editor. Reptile Medicine and Surgery 2nd edition. St. Louis, MO: Saunders Elsevier (2006). p. 631–64. 10.1016/B0-72-169327-X/50040-7

[31] Silverstein D Santoro-Beer K. Intravenous fluid therapy. In: Silverstein D, Hopper K, editors. Small Animal Critical Care Medicine. St. Louis, MO: Saunders Elsevier (2014). p. 316–20. 10.1016/B978-1-4557-0306-7.00059-3

[32] Parkinson LA Mans C. Evaluation of subcutaneously administered electrolyte solutions in experimentally dehydrated inland bearded dragons (*Pogona vitticeps*). Am J Vet Res. (2020) 81:437–41. 10.2460/ajvr.81.5.437 32343174

[33] Emmett M. Metabolic alkalosis: A brief pathophysiologic review. Clin J Am Soc Nephrol. (2020) 15:1848–56. 10.2215/CJN.16041219 32586924 PMC7769018

